# *Salvia przewalskii* extract of total phenolic acids inhibit TLR4 signaling activation in podocyte injury induced by puromycin aminonucleoside *in vitro*

**DOI:** 10.1080/0886022X.2018.1456460

**Published:** 2018-04-05

**Authors:** Hongqi Ren, Xueqing Hu, Yun Liu, Deshu Dai, Xiang Liu, Zenghui Wang, Yang Yang, Xiangyang Li, Ying Liu, Renxian Tang

**Affiliations:** a Department of Nephrology, The 97th Hospital of PLA Affiliated with Bengbu Medical College, Xuzhou, China;; b Department of Nephrology, Huaihai Hospital Affiliated with Xuzhou Medical University, Xuzhou, China;; c Department of Pharmacy, Huaihai Hospital affiliated with Xuzhou Medical University, Xuzhou, China;; d Department of Laboratory of Infection and Immunity, Xuzhou Medical University, Xuzhou, China

**Keywords:** Podocyte, puromycin aminonucleoside, *Salvia przewalskii* extract of total phenolic acids, salvianolic acid B, rosmarinic acid, TLR4

## Abstract

**Background:** TLR4 signaling is known to be involved in podocyte injury. We have previously shown that *Salvia przewalskii* extract of total phenolic acids (SPE) and its active monomer salvianolic acid B (SalB) and rosmarinic acid (RA) protect podocytes from injury induced by PAN. In the present study, we test whether SPE inhibits TLR4 signaling.

**Methods:** The conditionally immortalized mouse podocytes were treated with SPE, SalB, RA, SalB + RA or tacrolimus for 30 min, followed by PAN (100 μg/mL) for 24 h. The F-actin staining with phalloidin was used to assess cytoskeletal injury in the podocytes. Western blotting and semi-quantitatives RT-PCR were used to assess the changes of the components in the TLR4 signaling pathway.

**Results:** (1) The F-actin stress fibers of podocytes were almost completely disrupted after PAN treatment for 24 h, and the disruption was significantly alleviated by SPE; (2) the PAN-induced elevation of mRNA levels of TLR4, MyD88 and p65 were inhibited except p65 with high-dose SalB; (3) consistently, the protein levels of TLR4, MyD88 and pp65 were significantly elevated by PAN, and SPE, SalB, RA and admixture, respectively, attenuated the elevations of TLR4 and pp65 proteins; (4) SPE and tacrolimus have a similarly strong effect on inhibition of the expression of TLR4 signaling components.

**Conclusions:** SPE protects podocytes from PAN-induced injury at least partly through inhibiting TLR4 signaling. SPE is as strong as tacrolimus in inhibiting TLR4 signaling in podocytes.

## Introduction

*Salvia przewalskii* Maxim. is a perennial herb plant of the Labiatae family of flowering plants. The roots and rhizomes of this plant have been used as herbal remedies in traditional Chinese medicine and have the effects of regulating menstruation, activating blood circulation, eliminating stasis and relieving pain [1,2]. *Salvia przewalskii* extract (SPE) can decrease blood viscosity and improve blood circulation in rats. It also has a mild diuretic effect on water load in rats [3,4].

Our previous study showed that SPE can reduce proteinuria, protect podocyte cytoskeletons and restore the expression of genes critical for podocyte structure and function in rats with nephrosis induced by PAN [5]. Our further study showed that SPE also attenuates the production of 8-OHdG in PAN nephrosis [6]. SPE and the monomers can reduce ROS production and prevent mitochondrial fission in PAN-treated podocytes. The antioxidant effect of high-dose SPE and SalB in PAN-treated cultured podocyte is through suppression of ROS production and mitochondrial fission. In addition, we also found that SPE, SalB and RA can suppress PAN-induced apoptosis in podocytes [6].

Puromycin aminonucleoside is capable of inducing podocyte injury, including cytoskeletal abnormality, and is commonly used as a model for the study of podocyte injury. It has been reported that PAN can upregulate TLR4 expression in podocytes and TLR4 signaling has been implicated in podocyte injury [7–10]. In this study, we investigated whether SPE and its active monomers protect podocytes from PAN-induced injury through inhibiting TLR4 signaling using cultured podocytes *in vitro*.

## Materials and methods

### Reagents

The reagenes used in current study include: PAN (Sigma, Chemical Co., St. Louis, MO, USA), SalB, RA (Shanghai Source Leaf, China), Tacrolimus (Astellas, Japan), fetal bovine serum (ExCell Biology, Shanghai, China), RPMI 1640 culture medium, penicillin and streptomycin solution, 0.5% Trypsin-EDTA (Gibco, USA), Phalloidin (Santa Cruz Biotechnology, Santa Cruz, CA, USA), RIPA Lysis buffer, β-actin antibody, protein buffer (Beyotime Biotechnology, Shanghai, China), NF-kB, TLR2 antibody (Cell Signaling Technology, Danvers, MA, USA), TLR4 antibody (Dr. de Wuhan, China), MyD88 antibody (Boosen, Beijing, China), phospho-NF-kB (pp65) antibody (Bioworld, China) and reverse transcription and PCR kit (Tiangen, Beijing, China).

### Preparation of SPE

SPE (Batch No. 20090901-b15) was provided by the Pharmaceutical Chemistry Laboratory of the 97th Hospital of People Liberation Army (PLA) affiliated to Bengbu Medical College (Xuzhou Jiangsu, China). SPE was prepared by using dried roots and rhizomes of *S. przewalskii* and further purified by macroporous adsorptive resin column chromatograph according to the method described [4]. The RA and SalB contents in this batch of SPE were 31.58% and 5.52% according to the HPLC analysis using the area normalization method [6]. The DMSO stock at a high concentration was diluted before use and the final DMSO concentration was lower than 0.1%.

### Podocyte culture

The immortalized mouse podocyte cell line was provided by the National Clinical Research Center for Kidney Diseases (Nanjing, Jiangsu). The mouse podocytes were grown in the RPMI 1640 medium containing 10% fetal bovine serum, 100 U/mL of penicillin and 100 mg/L streptomycin and 10 U/mL IFN-γ, at 33 °C with 5% CO_2_. The cells were then cultured at 37 °C and 5% CO_2_ for 10–14 days for phenotypic differentiation. Serum starvation was performed 12 h prior to treatment.

### Experimental design

The podocytes were divided into the groups of untreated control, PAN, PAN + SPE (high and low doses, respectively), SalB (high and low doses, respectively), RA (high and low doses, respectively), SalB + RA mixed (high and low doses, respectively) and positive control tacrolimus. The differentiated podocyte was pre-incubated with SPE, RA, SalB, RA + SalB or Tac for 30 min, followed by PAN (100 µg/mL) for 24 h. The drug concentrations in the high and low dose groups were: SPE, 316 and 158 μg/mL; SalB, 17 and 8.5 μg/mL; RA, 50 and 25 μg/mL, respectively; the tacrolimus, concentration in the positive control was 1 μg/mL as previously described [6].

### Phalloidin staining for F-actin

Fluorescence microscopy was used to evaluate the changes in cell structure and actin cytoskeleton. After treatment, the podocytes were washed three times with PBS prewarmed at 37 °C, and fixed with 4% paraformaldehyde for 10 min. The cells were then rinsed with PBS for three times, followed by permeabilization with 0.5% Triton X-100 for 5 min. The cells were then washed with PBS and incubated with Phalloidin (1:400, Santa Cruz Biotechnology) for 30 min in the dark at room temperature. Images were acquired using a fluorescence microscope (Olympus, Tokyo, Japan).

### Western blot

After treatment, cell lysis buffer containing PMSF and phosphatase inhibitor was added to the cells, and incubated on ice for 30 min. The cell lysate was then collected and centrifuged at 12,000 *g* for 15 min at 4 °C, and the supernatant was transferred to a fresh tube. The protein concentration of the lysate was determined by the BCA protein assay kit (Beyotime Biotechnology, China). A 50 μg of protein was mixed with SDS-PAGE loading buffer and boiled for 5 min, and was then loaded on to 10% SDS-PAGE separation gel and underwent electrophoresis. The proteins on the gel were transferred to PVDF membrane with a transfer apparatus. The membrane was incubated in 5% skimmed milk at room temperature for 2 h and incubated with anti-TLR4 polyclonal antibody (1:200; Boster, China), anti- MyD88 polyclonal antibody (1:300; BIOSS, China), anti-NF-κB polyclonal antibody (1:1000; Cell Signaling Technology, Danvers, MA, USA), anti-pp65 polyclonal antibody (1:500; Bioworld, China) and anti-beta-actin (1:1,000; Beyotime Biotechnology, China) overnight at 4 °C. Subsequently, the membrane was washed with TBST, and then incubated with the second antibody (1:5000, Santa Cruz Biotechnology, Santa Cruz, CA, USA) for 1 h at room temperature. The membrane was washed with TBST. Finally, the membrane was incubated with the highly sensitive ECL reagent (Bio-Rad Laboratories, Hercules, CA, USA), and exposed in the BIO-RAD Chemical Imaging System. The resulting images were analyzed with Image-J software (National Institutes of Health, Bethesda, MD, USA).

### Semi-quantitative RT-PCR

Total RNA was extracted using RNAiso Plus Reagent, and its purities and concentrations of the total RNA samples were determined using Biophotometer (provided by Eppendorf China Ltd., Germany). The cDNA was synthesized using a reverse transcription kit (TIANGEN Biotech, China). We determined the mRNA expression levels of TLR4, MyD88 and NF-kB by semi-quantitative RT-PCR. Primer sequences are listed in Table 1. We followed the manual instruction of the kit to perform the RT-PCR. Electrophoresis with 2% agarose gel was run to detect the products of the PCR, and the result was recorded by a gel imaging system and analyzed by Image-J software.

**Table 1.** The primer sequences.

Primer	Primer sequences		Annealing temperature (°C)
TLR4	Forward	5′-CAAGAACATAGATCTGAGCTTCAACCC	60
	Reverse	5′-GCTGTCCAATAGGGAAGCTTTCTAGAG	
MyD88	Forward	5′-AAGAAAGTGAGTCTCCCCTC	55
	Reverse	5′-TCCCATGAAACCTCTAACAC	
NF-κB	Forward	5′-GCTCGGCTGAATGAATCTACC	60
	Reverse	5′-GTCTCCACGTATTTCCGCAACT	
*β*-Actin	Forward	5′-GGGAAATCGTGCGTGACATTAAG	60
	Reverse	5′-TGTGTTGGCGTACAGGTCTTTG	

### Statistical analysis

Statistical analyzes were performed with SPSS 16.0 software (version 16.0, SPSS Inc., Chicago, IL, USA). The results were expressed as the mean ± SD. Statistical analysis of the differences between two groups of treatment was performed using variance analysis combined with the rank sum test. *p* < .05 was considered statistically significant.

## Results

### Assessment of the protective effect of SPE on podocyte cytoskeletons

**Figure 1.**
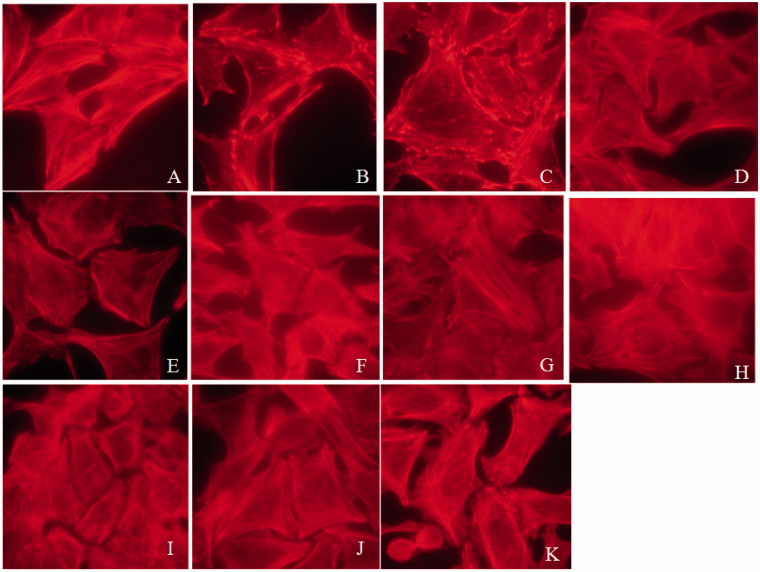
Tacrolimus, SPE, SalB and RA reversed PAN-induced podocyte actin cytoskeletal injury (× 1000). (A) Untreated normal control; (B) PAN; (C) SPE, 158 g/mL; (D) SPE, 316 g/mL; (E) RA, 25 g/mL; (F) RA, 50 g/mL; (G) SalB, 8.5 g/mL; (H) SalB, 17 g/mL; (I) SalB and RA, 8.5 + 25 g/mL combined; (J) SalB and RA, 17 + 50 g/mL combined; (K) positive drug control.

To assess the protective effects of SPE on podocytes, we first examined F-actin stress fibers in the PAN-treated cells without or with SPE reagents. We found that the F-actin stress fibers in the cells treated with PAN were reduced and disorganized obviously as shown by Phalloidin staining, accompanied by cell retraction. In contrast, in the presence of SPE, SalB, RA, SalB + RA or tacrolimus, the PAN-induced podocyte cytosekeletal injury was alleviated to varying degrees as shown by the restoration of typical pattern of F-actin stress fibers in the cells (Figure 1). These results demonstrated that SPE protects podocyte cytoskeletons from PAN-induced injury.

### Assessment of effect of SPE on TLR4 signaling activity by semi-quantitative RT-PCR

**Figure 2.**
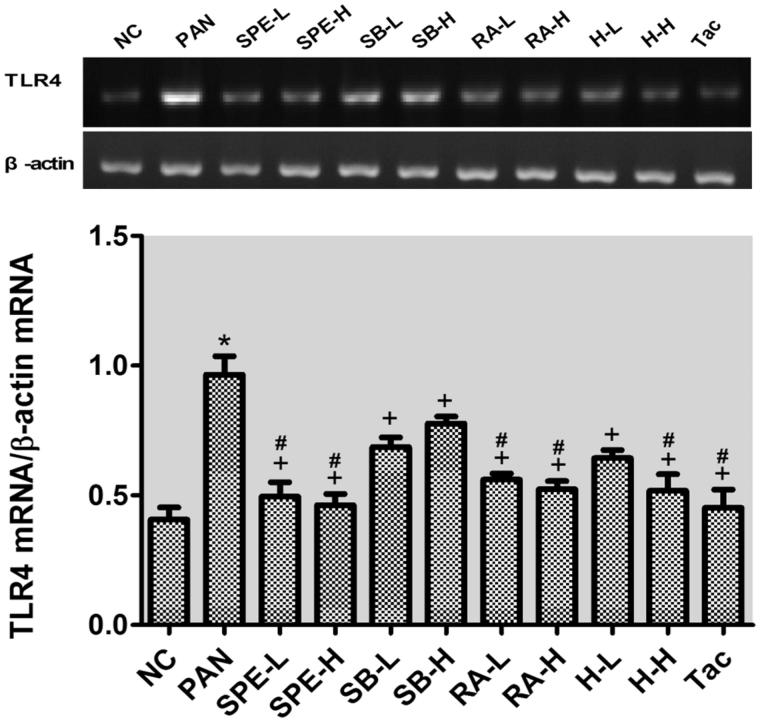
The effects of SPE and monomer on TLR4 mRNA levels in PAN-treated podocytes. NC, untreated control; PAN, model; SPE-L, 158 g/mL; SPE-H, 316 g/mL; SB-L, 8.5 g/mL; SB-H, 17 g/mL; RA-L, 25 g/mL; RA-H, 50 g/mL; H-L, SalB + RA (8.5 + 25 g/mL) combined; H-H, SalB + RA (17 + 50 g/mL) combined; Tac. positive control. Note: *p < .05 vs. NC; +*p* < .05 vs. PAN; #*p* > .05 vs. NC

**Figure 3.**
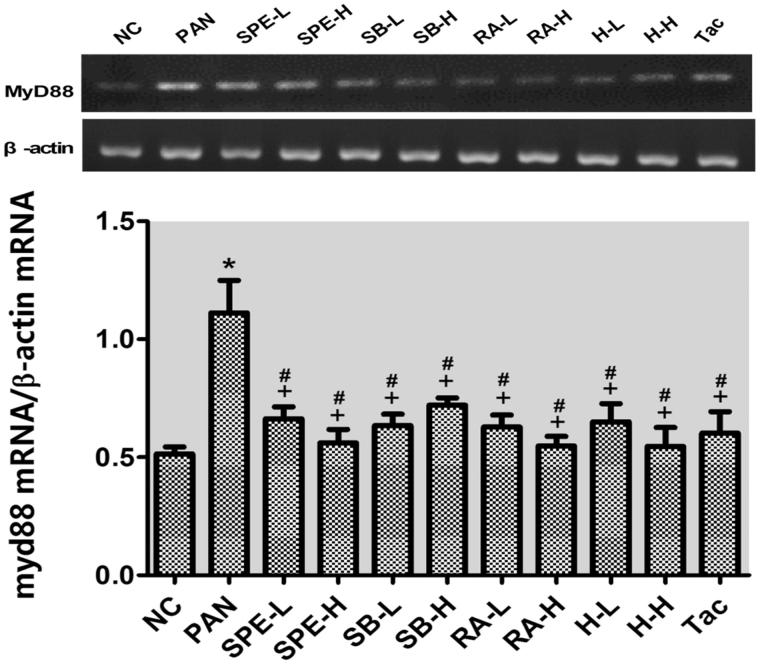
The effects of SPE and monomers on MyD88 mRNA levels in PAN-treated podocytes. NC, untreated control; PAN, model; SPE-L, 158 g/mL; SPE-H, 316 g/mL; SB-L, 8.5 g/mL; SB-H, 17 g/mL; RA-L, 25 g/mL; RA-H, 50 g/mL; H-L, SalB + RA (8.5 + 25 g/mL) combined; H-H, SalB + RA (17 + 50 g/mL) combined; Tac. positive control. Note: *p < .05 vs. NC; +*p* < .05 vs. PAN; #*p* > .05 vs. NC.

**Figure 4.**
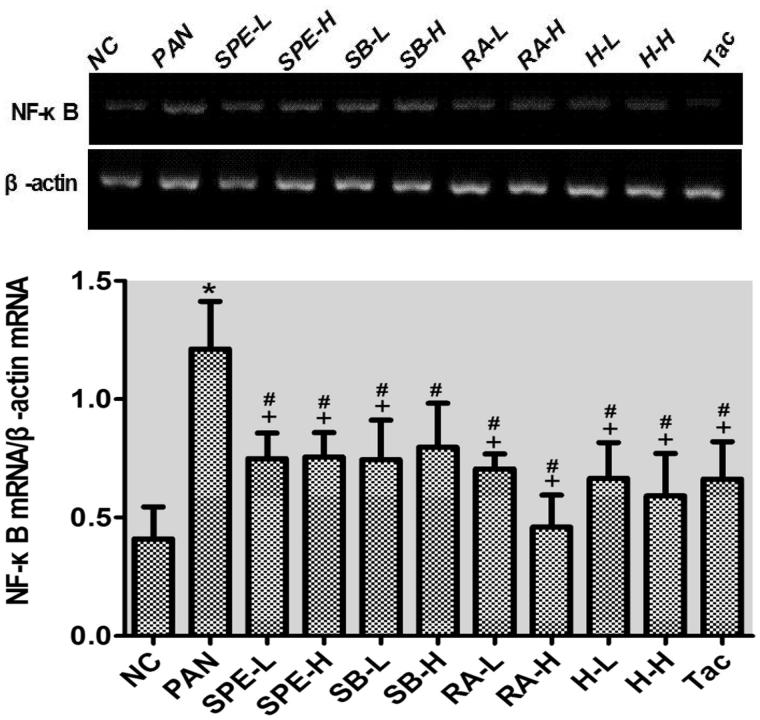
The effects of SPE and monomers on NF-kB mRNA level in PAN-treated podocytes. NC, untreated control; PAN, model; SPE-L, 158 g/mL; SPE-H, 316 g/mL; SB-L, 8.5 g/mL; SB-H, 17 g/mL; RA-L, 25 g/mL; RA-H, 50 g/mL; H-L, SalB + RA (8.5 + 25 g/mL) combined; H-H, SalB + RA (17 + 50 g/mL) combined; Tac. positive control. Note: *p < .05 vs. NC; +*p* < .05 vs. PAN; #*p* > .05 vs. NC

As mentioned earlier, we speculated SPE may protect podocytes through inhibiting TLR4 signaling. To test this hypothesis, we performed semi-quantitative RT-PCR to examine the mRNA expression of several TLK4 signaling components. The mRNA levels of TLR4, MyD88 and NF-kB in the PAN group were significantly higher than those in the normal control group (*p* < .05). In the drug intervention groups, the PAN-induced upregulations of TLR4 and MyD88 were prevented in all the groups to different degrees compared with the PAN group (Figures 2 and 3, *p* < .05). The NF-kB increases were also attenuated in those groups (Figure 4, *p* < .05) except the high-dose SalB group; particularly, TLR4 increases in the RA high- and low-dose groups, the high- and low-dose SPE groups, the high-dose SalB + RA group and the Tac group were attenuated most significantly, and its mRNA levels were close to normal (Figure 2, *p* > .05). Moreover, there were no significant differences in the MyD88 mRNA level between the intervention groups and the untreated normal group (Figure 3, *p* > .05). In addition to the high-dose RA group, NF-kappa B mRNA levels in the other intervention groups did not differ significantly from that of the normal group (Figure 4, *p* > .05). These results suggest that SPE can inhibit TLR4 signaling induced by PAN.

### Assessment of effect of SPE on TLR4 signaling activity by immunoblotting

**Figure 5.**
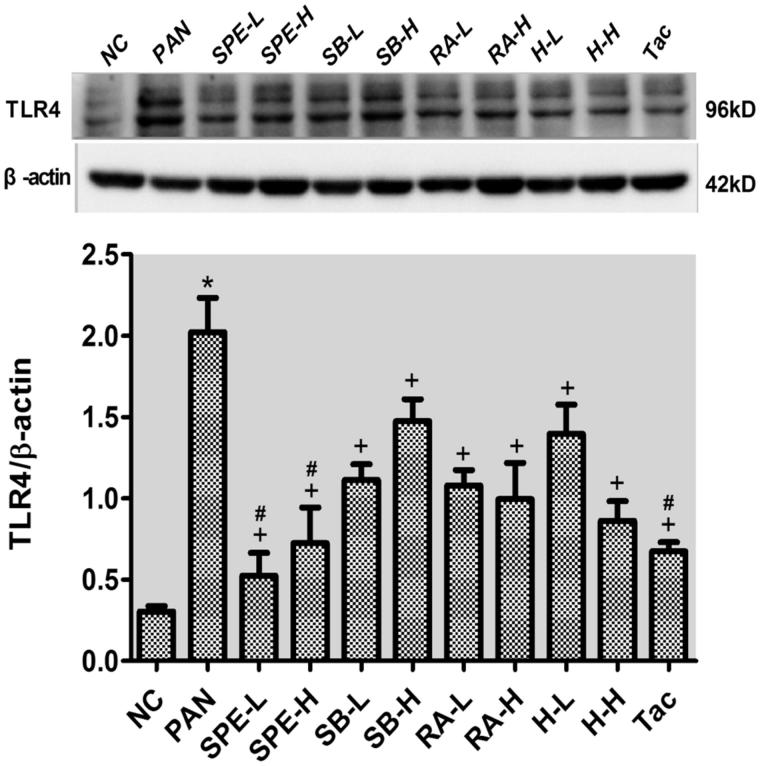
The effects of SPE and its monomers on the expression of TLR4 in PAN-treated podocytes. NC, untreated normal control; PAN, injury model; SPE-L, 158 g/mL; SPE-H, 316 g/mL; SB-L, 8.5 g/mL; SB-H, 17 g/mL; RA-L, 25 g/mL; RA-H, 50 g/mL; H-L, SalB + RA (8.5 + 25 g/mL) combined; H-H, SalB + RA (17 + 50 g/mL) combined; Tac. positive control. Note: *p < .05 vs. NC; +*p* < .05 vs. PAN; #*p* > .05 vs. NC

**Figure 6.**
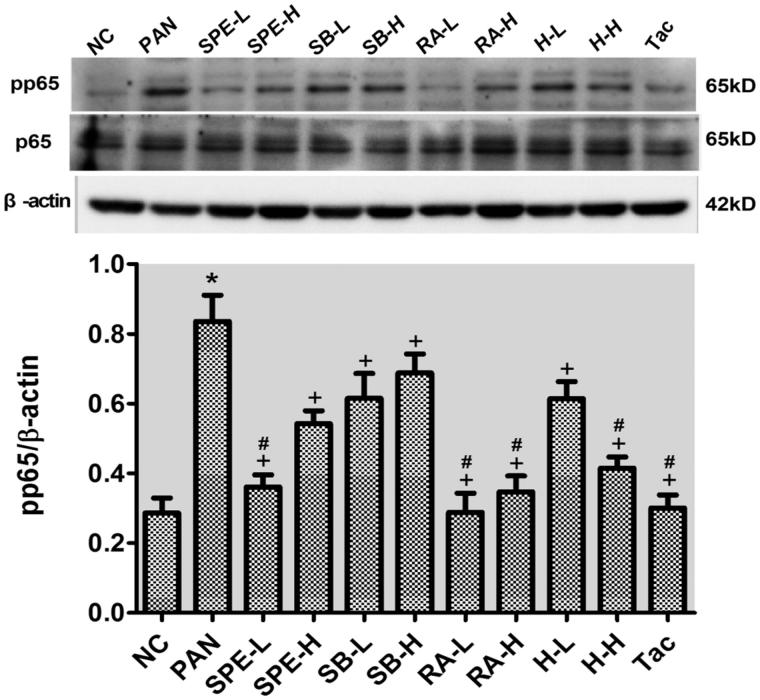
The effects of SPE and its monomers on the level of pp65 in PAN-treated podocytes. NC, untreated control; PAN, model; SPE-L, 158 g/mL; SPE-H, 316 g/mL; SB-L, 8.5 g/mL; SB-H, 17 g/mL group; RA-L, 25 g/mL; RA-H, 50 g/mL; H-L, SalB + RA (8.5 + 25 g/mL) combined; H-H, SalB + RA (17 + 50 g/mL) combined; Tac. positive control. Note: *p < .05 vs. NC; +*p* < .05 vs. PAN; #*p* > .05 vs. NC

**Figure 7.**
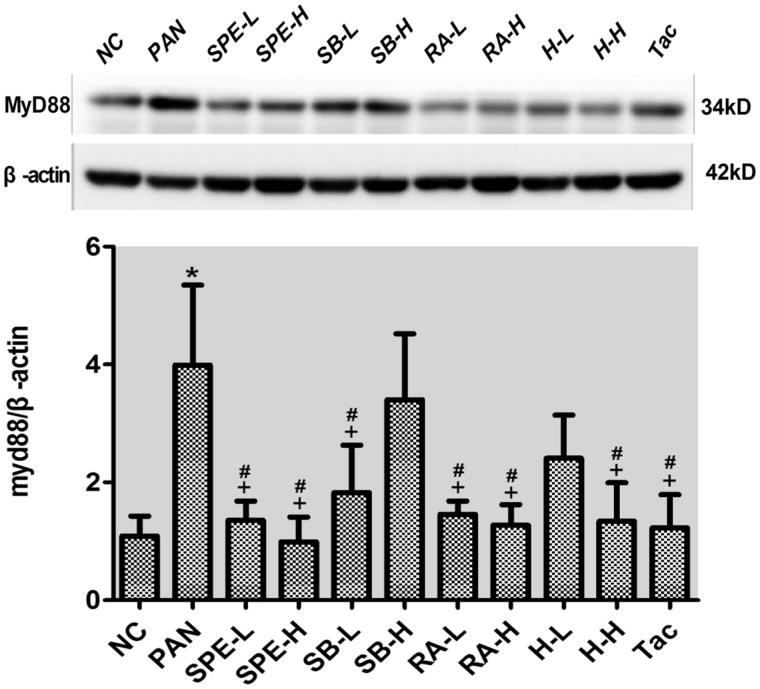
The effects of SPE and its monomers on the expression of MyD88 in PAN-treated podocytes. NC, untreated normal control; PAN, injury model; SPE-L, 158 g/mL; SPE-H, 316 g/mL; SB-L, 8.5 g/mL; SB-H, 17 g/mL; RA-L, 25 g/mL; RA-H, 50 g/mL; H-L, 8.5 + 25 g/mL combined; H-H, SalB + RA (17 + 50 g/mL) combined; Tac. positive control. Note: *p < .05 vs. NC; +*p* < .05 vs. PAN; #*p* > .05 vs. NC

To confirm that the expression of TLR4 signaling components can be inhibited by SPE, we additionally performed immunoblotting to examine the proteins. Compared with the normal control cells, the protein expressions of TLR4, the downstream adaptor protein MyD88 and the phosphorylated NF-kB p65 were increased in the PAN-treated cells. However, the PAN-induced increases of both TLR4 and pp65 were attenuated in the cells of all intervention groups (Figures 5 and 6, *p* < .05). The increased expression of MyD88 protein was also attenuated in the intervention groups except the groups of high-dose SalB and low-dose SalB + RA (Figure 7, *p* < .05). The PAN-induced increases of TLR4 protein in the cells of the high- and low-dose SPE groups, as well as the Tac control group, were almost completely prevented compared with that in the normal control group (Figure 5, *p* > .05). Similarly, the expression of MyD88 protein in the SPE high- and low-dose groups, the SalB low-dose group, the RA high- and low-dose group and the high- and low-dose SalB + RA groups, also returned to the level of the normal group (Figure 7, *p* > .05). Additionally, the PAN-induced increase of pp65 in the cells of the groups of low-dose SPE, high- and low-dose RA, high-dose SalB + RA and Tac were also mostly prevented (Figure 6, *p* > .05). These results confirmed that SPE can inhibit PAN-induced TLR4 signaling in podocytes.

## Discussion

We have previously shown that the extract of *S. przewalskii* Maxim. can protect podocyts from PAN-induced injury in rats [5], PAN nephropathy is a classic animal model for podocyte injury, including cytoskeletal damage [11]. It has been shown that PAN injurious effect is mediated by oxidative stress and other signalings, e.g., Notch [12–16], and previously we did show that SPE can inhibit PAN-induced oxidative stress in podocytes [6]. However, the exact mechanism underlying SPE protective effect required further investigation.

TLR4, a member of the TLR family, is able to recognize the lipopolysaccharides from gram-negative bacillus and trigger signaling for inflammatory response in the cells. Recent studies have shown that TLR4-specific inhibitor GIT27 or TLR4 knockout can alleviate proteinuria and podocyte injury, and prevent the increase of proinflammatory cytokine production in diabetic mice [9,10]. Previous studies have shown that PAN can upregulate TLR4 expression and the increased TLR4 expression can aggravate podocyte injury [7,8].

Our previous study has shown that SPE can reduce proteinuria, protect podocytes and alleviate the oxidative stress in the PAN-induced injury. The main active components of the SPE are RA and SalB [5,6]. Studies have shown that SalB can scavenge oxygen free radicals and inhibit lipid peroxidation [17], while RA has a strong anti-inflammatory and immunomodulatory effects in addition to its antioxidant effect [18]. These studies collectively suggest that SPE and its active monomers may prevent the activation of PAN-induced TLR4 signaling, thereby preventing podocyte injury. Thus, in the present study, we examined the effects of SPE and its active monomers on expression of the components in the TLR4 pathway in cultured mouse podocytes treated with PAN to test our hypothesis.

As expected, we observed that PAN upregulated the expressions of TLR4, MyD88 and activated NF-kB p65. These effects of PAN were abolished completely or at least partially by SPE or its active monomers. For example, the increases of TLR4 signaling components were prevented in the high- and low-dose SPE groups. We also observed that the inhibitory effect of monomeric RA was more prominent than that of SalB; however, interestingly the combined treatment of RA and SalB had a reduced inhibitory efficacy compared with RA, suggesting that the pathways that these two monomers act on could be distinct and antagonize each other. The detail of their interaction warrants further investigation.

Besides examining the expression of TLR4 signaling components, we also observed that SPE and its active monomers protected podocyte cytoskeletons, which was expected. This observation suggests that SPE and its active monomers protect podocyte cytoskeleton through inhibiting TLR4 signaling.

We used tacrolimus as positive control in the study because it is a calcineurin inhibitor (CNI), which can act on podocyte directly to stabilize their cytoskeletons and reduce proteinuria of animals [19–23]. In addition, tacrolimus has been shown to act on renal macrophages, downregulating the expression of TLR2 and TLR4 and inhibiting the inflammatory response through TLR-NF-kB signaling pathway [24]. Therefore, we used tacrolimus as a positive control to assess the inhibitory effect of SPE and its monomer on TLR4 signaling in the podocytes treated with PAN. Our results showed that SPE or its active monomers can be as effective as tacrolimus in inhibiting the expression of TLR4 signaling components, and they are a great potential to be used to treat various podocytopathies.

One limitation of this study is that we have only investigated TLR4 signaling pathway for elucidation of the protective effects of SPE and its active monomers. However, it is possible that the other TLR family members or other distinct pathways are also involved in their protective effects on PAN-induced podocyte injury. Further work to address this issue is warranty.

## Conclusions

This study has demonstrated that the protective effect of SPE and its active monomers on PAN-induced podocyte injury is partially mediated by their inhibition of TLR4 signaling. The inhibitory effect of RA on TLR4 signaling molecules is stronger than that of SalB and combined SalB and RA. In addition, SPE and the active monomers could achieve similar strength as tacrolimus in inhibiting TLR4 components expression, and are promising in the treatment of podocytopathies.
